# Physical activity in rheumatoid arthritis—is it time to push the pace of change?

**DOI:** 10.1093/rap/rkac107

**Published:** 2023-01-24

**Authors:** Lindsay M Bearne

**Affiliations:** Population Health Research Institute, St George’s, University of London, London, UK

Being physically active improves health outcomes in people with RA [1]. It is a recommended but often overlooked therapy for people with RA [2, 3]. Consequently, optimizing the role of physical activity (PA) in RA management is an important treatment ambition.

PA is defined as any bodily movement produced by skeletal muscles that requires energy expenditure beyond resting expenditure [4]. This encompasses activity related to work or transportation, in addition to leisure-time activity, such as play, games or sports. Planned, structured exercise to improve or maintain at least one dimension of physical fitness is a subcategory of PA. Therapeutic exercises are prescribed by health or fitness professionals to prevent or reduce functional problems or health-related risk factors, or to restore or optimize activity and participation [5, 6].

PA guidance for the general population includes ≥150 min of moderate-intensity PA or equivalent per week and strength training at least twice a week. In addition, people with long-term disabilities are recommended to complete multicomponent balance and strength training to enhance function [7]. EULAR endorses these recommendations for people with inflammatory arthritis. People with RA should aim to achieve these recommendations whilst accounting for their current activity level, disease activity and symptoms [3, 6]. Therapeutic exercise should include an individualized, progressive programme of moderate- to high-intensity aerobic training and resistance training of all muscle groups, and individuals with pain or dysfunction in the wrist and hands are recommended to complete a tailored strengthening and stretching exercise programme [2].

But people with RA have lower levels of PA than the general population [8] and tend not to reach recommended PA levels [8–11]. Barriers to PA include the disease, resource, social and environmental factors [12, 13]. Physical distancing measures imposed during the coronavirus disease 2019 (COVID-19) pandemic disproportionately affected PA levels adversely in people with RA owing to disrupted access to specialist exercise equipment and facilities, despite rapid transformation of health-care delivery [14–16].

Beyond the COVID-19 pandemic there is an opportunity for learning and reflection on the role and delivery of PA. In this context, this special issue is timely and important. It provides up-to-date syntheses of the evidence and shines a light on the challenges and new avenues to help us understand and optimize the role of PA in the management of RA.

## Equitable access to PA

Everyone with RA should be able to access appropriate and acceptable PA interventions to gain the health benefits. Canning *et al.* [17] poignantly describe their experiences of integrating PA into their lives. They provide examples of how they have adapted PA through their lives and how they coped with the challenge of maintaining PA participation during the COVID-19 pandemic. Although remotely delivered PA interventions are promising [16], evidence of effectiveness is limited [18, 19], and a personalized, person-centred approach to development of PA interventions that is guided by patient experience is needed.

Commissioning decisions for PA services are informed by evidence of clinical and cost effectiveness from randomized clinical trials or systematic reviews of trials, hence it is vital that trial participants are representative of the people who will use the intervention. Although the cost utility of PA interventions is largely unknown, predominantly home-based interventions and interventions directed at more severely affected people seem most cost effective at a societal level [20–22]. However, only a small proportion of people with RA screened for eligibility are reported to take part in exercise trials [23]. People with similar needs might not be equally able to take part. These inequities might be avoidable and unfair. Jenkins *et al.* [24] apply the PROGRESS-plus guidance framework [25] to review the eligibility criteria of randomized exercise trials to summarize the equity factors that influence the chance to participate in exercise interventions. All included trials excluded potential participants based on at least one equity factor, but few studies justified these decisions. Although researchers strive to enrol a representative sample of people with RA in exercise trials, uncertainty about the safety of new PA interventions might lead unintentionally to inequities in access to PA interventions.

## Understanding and capturing the role of PA

Understanding the combined effects of exercise and RA on micro-ribonucleic acids (miRNA) might help clinicians to tailor PA interventions and improve disease management. Balchin *et al.* [26] take a fresh look at the role of miRNAs in the regulation of inflammation and exercise-induced adaptations in people with RA. They suggest that acute exercise changes the levels of miRNAs commonly associated with disease progression in RA. The exercise dose, but also individual variation, affects miRNAs levels after an exercise session. It might be that miRNAs regulate several of the adaptations induced by exercise, including muscle hypertrophy, cardiovascular fitness and angiogenesis.

Measurement is key to evaluating the usefulness of PA interventions. The estimation of energy expenditure (called metabolic equivalents of tasks) in controlled laboratory environments and assessment of activity patterns in free-living environments are reviewed by Steultjens *et al.* [27]. Body-worn sensors currently offer the best option to assess activity patterns in free-living environments, although implementation into clinical practice might be challenging. Devices that complement accelerometery with other measures (e.g. heart rate) might improve the measurement properties of these tools.

## Helping people with inflammatory arthritis to move more

Sedentary behaviour (waking activities in a seated or reclining posture, requiring ≤1.5 metabolic equivalents of tasks) is common in people with RA and ranges between 8.3 and 14.0 h/day [8]. Minimizing sedentary behaviour provides health benefits and might offer another promising management approach for people with RA [8, 28, 29]. Fenton *et al.* [30] draw on the five-phase behavioural epidemiology framework to summarize the current evidence on the role and mitigation of sedentary behaviour in people with inflammatory arthritis [31]. They highlight the sporadic nature and mixed quality of research targeting sedentary behaviour, gaps in our understanding, and a lack of causal, experimental research to inform effective intervention approaches. A systematic approach to future investigations is recommended [30].

The challenge of supporting people to be physically active is well recognized, and the EULAR recommendations for PA for people with inflammatory arthritis suggest that behavioural change techniques should be integrated into interventions to support adherence [3]. A scoping review by Chaplin *et al.* [32] reveals that adherence to PA was seldom the focus of research studies and that evidenced-based interventions are lacking. Some interventions that targeted adherence to exercise did not use psychological theory to inform the intervention design, include evidence-based behavioural change techniques or use validated measures of adherence to evaluate the effects interventions. Further investigation of the determinants of PA adherence and the development of targeted interventions to enhance adherence to PA in people with RA are needed. It might be that we should look beyond the health-care system to support PA. Social prescribing is one approach that involves empowering link workers (also known as community navigators) to help people find local, non-clinical opportunities to help them stay active.

Even when effective and cost-effective PA interventions are available, there are delays in translating these interventions into practice. Cornwall *et al.* [33] draw attention to the multilevel issues that might influence the scale up and spread of PA interventions, including individual (public and professional), organizational and policy factors. Considering knowledge mobilization and implementation strategies, adopting an implementation framework and engaging members of the public and professional groups to identify and mitigate barriers to implementation early in the intervention and study design might help to address this implementation gap.

I am grateful to all the contributors to this supplement for their careful review of the evidence and forward-thinking approach to the role of PA. This illustrates that it is time to innovate and push the pace of change to optimize the delivery and use of this overlooked management strategy.

## Data Availability

No new data were generated or analysed in support of this research.
